# Expression and localisation of Rab44 in immune-related cells change during cell differentiation and stimulation

**DOI:** 10.1038/s41598-020-67638-7

**Published:** 2020-07-01

**Authors:** Mitsuko Tokuhisa, Tomoko Kadowaki, Kohei Ogawa, Yu Yamaguchi, Mizuho A. Kido, Weiqi Gao, Masahiro Umeda, Takayuki Tsukuba

**Affiliations:** 1 Department of Frontier Life Science, Graduate School of Biomedical Sciences, Nagasaki University, Sakamoto 1-7-1, Nagasaki, 852-8588 Japan; 2 Department of Dental Pharmacology, Graduate School of Biomedical Sciences, Nagasaki University, Sakamoto 1-7-1, Nagasaki, 852-8588 Japan; 3 Department of Clinical Oral Oncology, Nagasaki University Graduate School of Biomedical Sciences, Nagasaki, Sakamoto 1-7-1, Nagasaki, 852-8588 Japan; 4 Department of Anatomy and Physiology, Faculty of Medicine, Saga University, Saga, 849-8501 Japan

**Keywords:** Biochemistry, Cell biology, Developmental biology

## Abstract

Rab44 is a large Rab GTPase that contains a Rab-GTPase domain and some additional domains, such as EF-hand and coiled-coil domains at the N-terminus. Our previous study showed that Rab44 negatively regulates osteoclast differentiation by modulating intracellular calcium levels; however, aside from those findings, there is little information concerning Rab44 on other cells or tissues. In this study, we showed that Rab44 was highly expressed in bone marrow cells among various mouse tissues. Immunohistochemical studies indicated that Rab44 was detectable by only a small number of cells in the immune-related tissues and that Rab44 was partially detected in CD117-positive cells, but not in Stem cell antigen 1-positive cells in the bone marrow. Rab44 expression levels were decreased during differentiation of immune-related cells, such as neutrophils, macrophages, and dendritic cells compared with bone marrow cells. Although endogenous Rab44 in macrophages was localised in lysosomes, lipopolysaccharide (LPS) stimulation led to partial translocation to early endosomes and the plasma membrane. Moreover, Rab44 expression levels were altered by treatment with various immunomodulators, including LPS. These results indicate that Rab44 expression and localisation in bone marrow cells and macrophages alters with cell differentiation and stimulation.

## Introduction

Rab GTPases are critical regulators of intracellular membrane trafficking, including vesicle transport, membrane fission, tethering, docking, and fusion events^1,2^. Rab GTPases coordinate membrane trafficking as molecular switches that change conformational states between active GTP-bound and inactive GDP-bound forms^3^. At present, there are 66 Rab genes in the human genome^4,5^. Each Rab GTPase localises to a distinct membrane compartment to modulate membrane trafficking. Among various Rab GTPases, Rab1, Rab5, Rab6, Rab7, and Rab11 are known as ‘housekeeping Rabs’, since they are conserved from yeast to humans^6^. Meanwhile, most other Rabs have unique cell type-specific or tissue-specific roles. For example, Rab3 and Rab27 members are termed as ‘secretory Rabs’ that are predominantly localised in neurons and endocrine cells that have unique vesicles for regulatory secretion^7^. In contrast to these well-characterised Rabs, the cellular function of Rab44 is poorly investigated.

Rab44 is a large Rab GTPase that encodes several domains, such as the EF-hand domain, coiled-coil domain, and Rab-GTPase domain^8^. The amino acid sequences of human Rab44 indicate a putative molecular mass of approximately 110 kDa. Considering that Rab 1–43 are the monomeric small GTPases with molecular weights of about 20–30 kDa, Rab44 is an atypical Rab GTPase of approximately 75–150 kDa. Recently, our research group has discovered that Rab44 expression is transiently upregulated during osteoclast differentiation^9^. Moreover, knockdown of Rab44 promotes osteoclast differentiation, whereas overexpression of Rab44 prevents it. Rab44 overexpressed in macrophages is predominantly localised in the Golgi complex and lysosomes, and Rab44 causes an enlargement of early endosomes. Mechanistically, it is likely that Rab44 affects nuclear factor of activated T-cells c1 (NFATc1) signalling in RANKL-stimulated macrophages via an elevation in lysosomal calcium influx. These results suggest that Rab44 negatively regulates osteoclast differentiation by controlling intracellular calcium levels followed by NFATc1 activation. However, except for the findings regarding the effect of Rab44 on osteoclast differentiation, there is little information concerning Rab44 on other cells or tissues.

In this study, we examined tissue distribution, expression, and localisation of mouse Rab44. We showed that endogenous Rab44 is highly expressed in bone marrow cells and that Rab44 expression was changed during the differentiation of immune-related cells and by treatment with immunomodulators.

## Results

### Rab44 is expressed highly in the bone marrow and weakly in immune-related tissues

**Figure 1:**
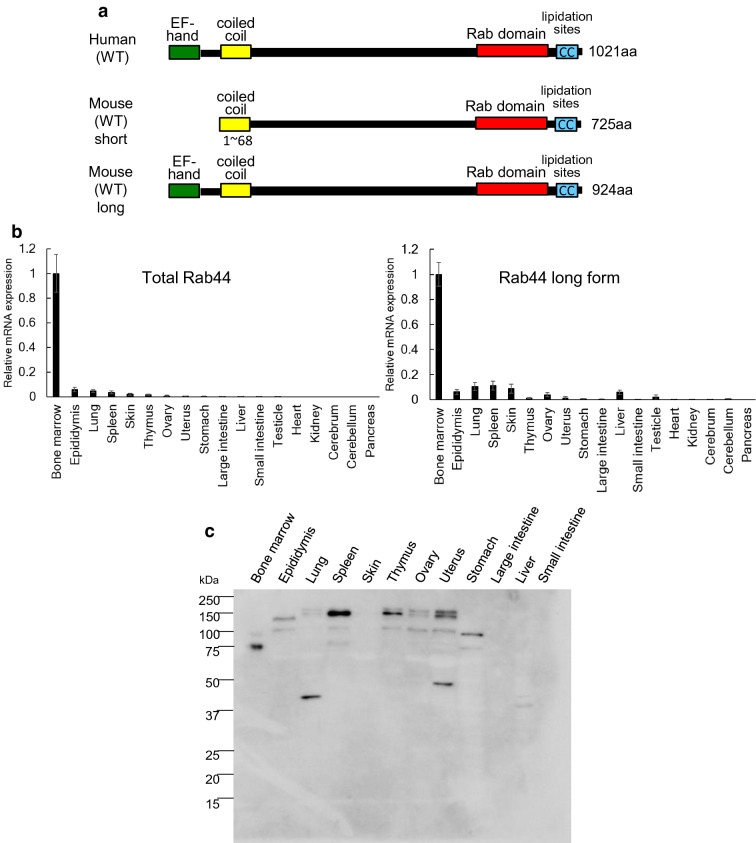
Tissue distribution and expression of Rab44 in mice. (**a**) Schematic representation of transcripts of mouse Rab44 and human Rab44. (**b**) Quantitative RT-PCR analysis of Rab44 mRNA expression levels in various mouse tissues. The data show the relative expression levels of short- and long-form mouse Rab 44 compared to that of the bone marrow as the control. The data are represented as mean ± S.D. of values from three independent experiments. (**c**) Western blotting of Rab44 protein expression levels in various mouse tissues. Cell lysates were subjected to SDS-PAGE followed by western blotting with antibodies against Rab44.

Our previous study showed that human Rab44 encodes an N-terminal EF-hand domain, a mid-regional coiled-coil domain, and a C-terminal Rab-GTPase domain, while mouse Rab44 lacks the N-terminal EF-hand domain^9^. However, during several experiments, we found that mouse Rab44 also contains all three above mentioned domains^10^. Therefore, we termed Rab44 containing the N-terminal EF-hand domain as ‘long form’ and Rab44 lacking this domain as ‘short form’ (Fig. 1a).

We first analysed the tissue distribution and expression levels of both forms of mouse Rab44. Then, we separately measured the total amount (sum of long and short forms) of Rab44 and the amount of long-form Rab44 in various mouse tissues using quantitative real-time polymerase chain reaction (RT-PCR) analysis. At the mRNA level, both forms of Rab44 were highly expressed in the bone marrow and slightly expressed in the epididymis, lung, skin, spleen, thymus, ovary, uterus, and liver (Fig. 1b). Rab44 expression was hardly detected in the stomach, small intestine, large intestine, heart, kidney, cerebrum, cerebellum, and pancreas (Fig. 1b).

To examine Rab44 protein expression, western blotting was performed. The Rab44 proteins in the bone marrow were detected as two forms with molecular masses of approximately 75 and 100 kDa (Fig. 1c). However, the Rab44 proteins from the spleen and thymus were detected as having molecular masses of approximately 150 kDa. Since these bands were not detected in the bone marrow or other tissues of Rab44 knockout mice, we confirmed that these bands were Rab44 specific (data not shown).

### Rab44 is detectable by only a small number of cells in the immune-related tissues

**Figure 2:**
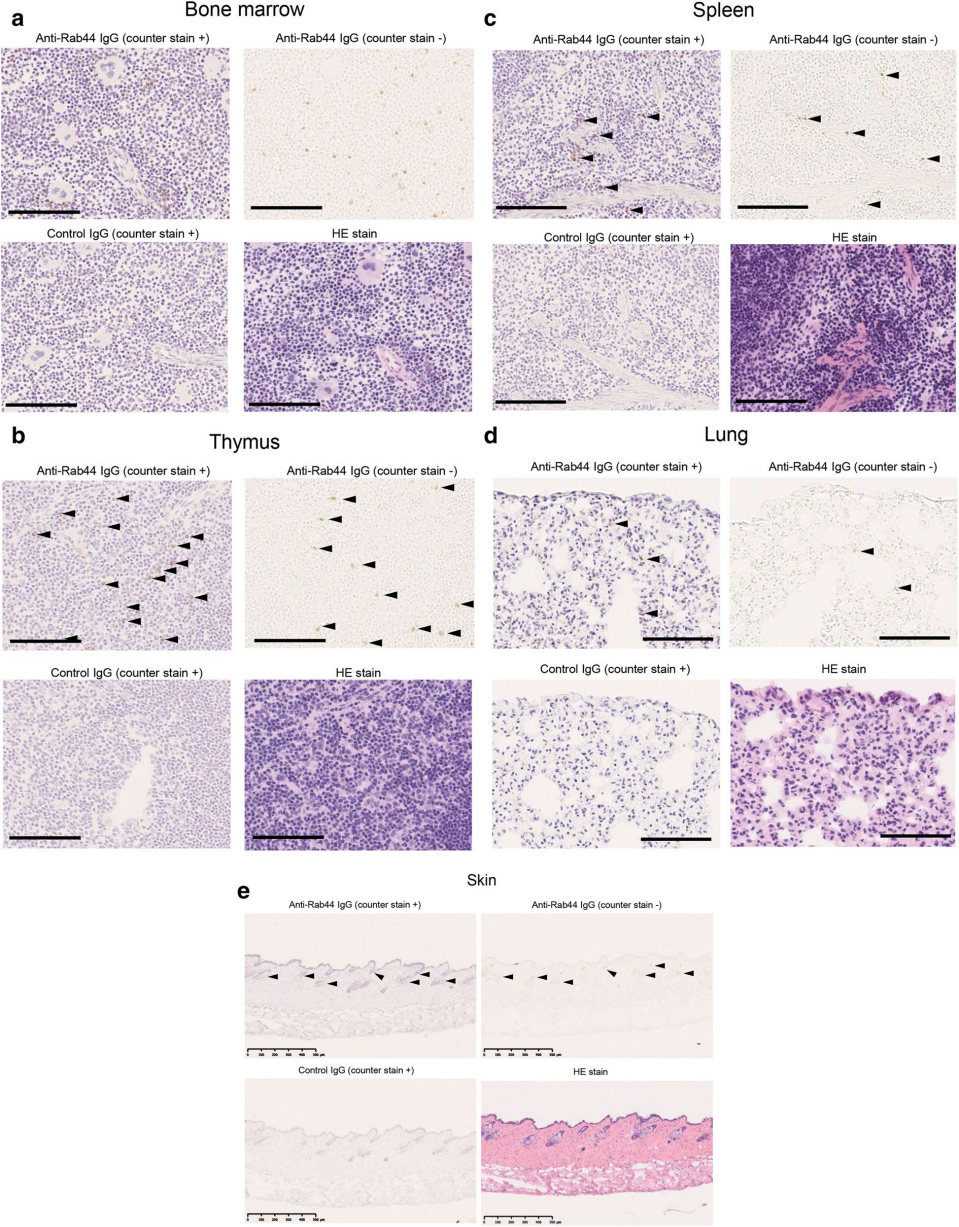
Immunohistochemical analysis of Rab44 expression in tissues. Upper left, immunostaining with anti-Rab44 antibody and counterstaining; upper right, immunostaining with anti-Rab44 antibody only; lower left, immunostaining with control IgG and counterstaining; lower right, counterstaining with haematoxylin and eosin stain only. Bars: 100 μm. (**a**) Bone marrow, (**b**) thymus, (**c**) spleen, (**d**) lung, (**e**) skin.

To explore the distribution of cells expressed in Rab44, we performed immunohistochemical analysis. The results in the bone marrow are shown in Fig. 2a. The Rab44-positive cells were sparsely detected throughout the marrow as a whole, although they were hardly detected in the megakaryocytes. As a negative control, the immunoreaction for non-specific IgG was hardly detected in the bone marrow (Fig. 2a). These results indicate that the Rab44-positive cells are present in a small portion of bone marrow cells.

We further examined Rab44-enriched tissues such as the thymus, spleen, lung, and skin. In the thymus, Rab44-positive cells were scattered throughout the tissue (indicated by the arrowhead) (Fig. 2b). There were many Rab44-positive cells in the medulla, but fewer in the cortex (Fig. 2b). Concerning the spleen, Rab44-positive cells were detected in the splenic cord in the red spleen (Fig. 2c). However, Rab44 was hardly detectable in the white pulp of many lymph nodes (Fig. 2c). In the lung tissue, weak immunoreactivity was sparsely detectable in relatively large cells (Fig. 2d). In the skin, the Rab44-positive cells were observed in the epidermis, subcutaneous fat layer, and sebaceous glands (Fig. 2e).

### Rab44 is extensively expressed in CD117^+^ cells, but not in Sca-1^+^ cells in haematopoietic cells in the bone marrow

**Figure 3:**
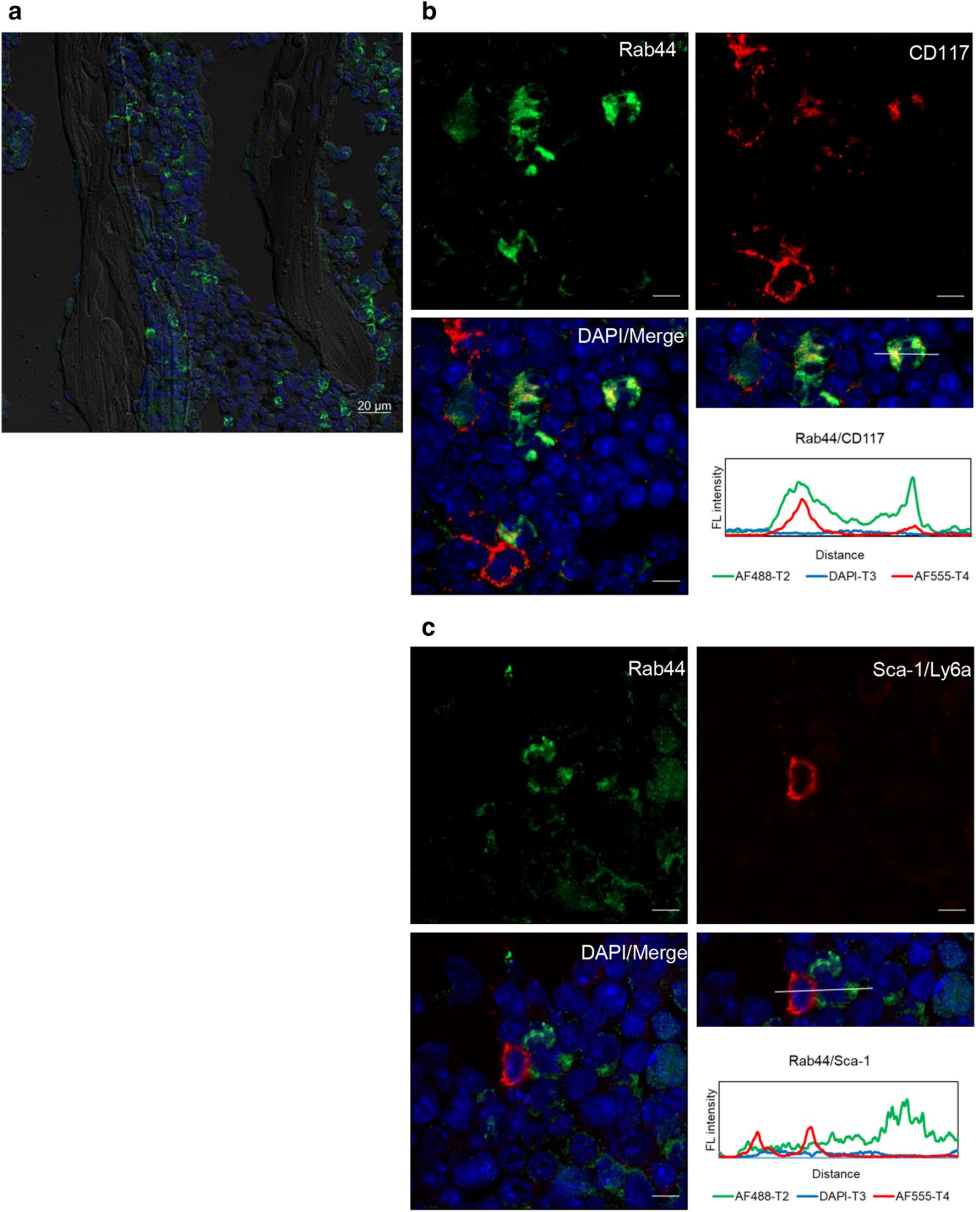
Immunofluorescence analysis of Rab44 and markers for haematopoietic stem cells in the bone marrow. (**a**) The fixed sections of femur containing bone marrow were blocked in PBS containing 5% normal donkey serum. The samples were incubated with rabbit polyclonal anti-Rab44 IgG (1:1,000) as the first antibody followed by fluorescent labelling with Alexa fluor 488-conjugated anti-rabbit IgG and then visualised by confocal laser microscopy. Bar: 20 μm. (**b**, **c**) The mouse bone was fixed, permeabilised with 0.1% Triton X-100 in PBS, and then allowed to react with antibodies against Rab44 and (**b**) CD117 or (**c**) Sca-1. After washing, the samples were incubated with a fluorescence-labelled secondary antibody and then visualised by confocal laser microscopy. Bar: 5 μm.

To further analyse the specificity of Rab44 in bone marrow cells, we performed fluorescent immunohistochemical staining (Fig. 3). Among many Rab44-negative cells, some strongly positive cells for Rab44 were detected (Fig. 3a). When we examined the relationship between Rab44 and markers for haematopoietic cells in the bone marrow, we found that Rab44 was partially merged with CD117 in the same cells (Fig. 3a). CD117 (c-kit) is a typical marker for undifferentiated haematopoietic cells and is a receptor tyrosine kinase that is expressed on haematopoietic stem cells (HSCs), mast cells, germ cells, and melanocytes. Rab44^+^/CD117^+^ double-positive cells were found, but Rab44^+^/CD117^−^ or Rab44^−^/CD117^+^ single-positive cells were also present in the bone marrow (Fig. 3a). In contrast, we observed that Rab44 failed to merge with Stem cell antigen-1 (Sca-1; Ly-6 A/E), which is a GPI-linked cell surface protein and a useful marker for stem cells, including haematopoietic, endothelial, mesenchymal, and cardiac progenitor cells (Fig. 3b). Rab44^+^ cells and Sca1^+^ cells were independently present in the bone marrow (Fig. 3b). These results indicate that Rab44 is detected in CD117^+^ cells, but not in Sca-1^+^ cells in the bone marrow.

### Rab44 expression levels were decreased during differentiation of immune-related cells

**Figure 4:**
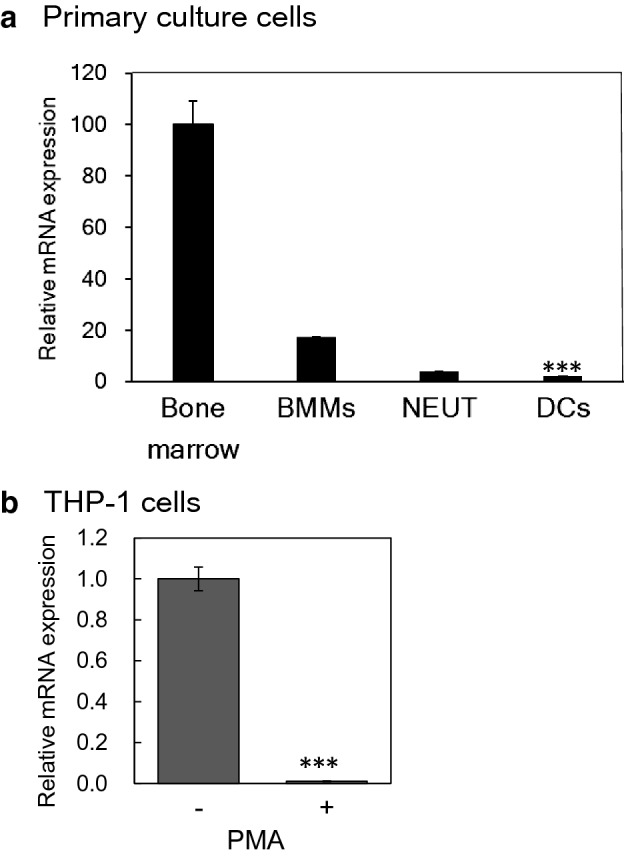
Comparison of expression levels of Rab44 in bone-marrow cells and immune-related cells. (**a**) Quantitative RT-PCR determination of Rab44 mRNA expression in mouse bone marrow cells, BMMs, NEUTs, and DCs. The data are represented as the mean ± S.D. of values from three independent experiments. (**b**) Quantitative RT-PCR determination of Rab44 mRNA expression in human THP-1 cells. THP-1 cells were incubated with PMA (30 nM) for 2 days. The data are represented as the mean ± S.D. of values from three independent experiments. The asterisks indicate statistical significance compared to the control cells without PMA treatment, ***P < 0.005.

Next, we analysed Rab44 expression levels in representative immune cells, such as bone-marrow-derived macrophages (BMMs), neutrophils (NEUTs), and dendritic cells (DCs) that differentiate from bone marrow cells (Fig. 4a). RT-PCR analysis revealed that the total amount of Rab44 expressed by BMMs was about 20% of that expressed by bone marrow cells (Fig. 4a). Similarly, Rab44 expression from NEUTs and DCs was about 5% and 3%, respectively (Fig. 4a). Thus, Rab44 levels in BMMs, NEUTs, and DCs were found to be extremely low compared to bone marrow cells.

Similar results were observed in a human monocytic leukaemia cell line, THP-1 (Fig. 4b). Stimulation of THP-1 cells with phorbol 12-myristate 13-acetate (PMA), which induce differentiation into macrophage-like cells, markedly reduced the expression of Rab44 (Fig. 4b). These results also indicate that Rab44 levels are commonly decreased in mouse and human macrophages during differentiation from their precursor cells.

### Endogenous Rab44 in BMMs is localised in lysosomes

**Figure 5:**
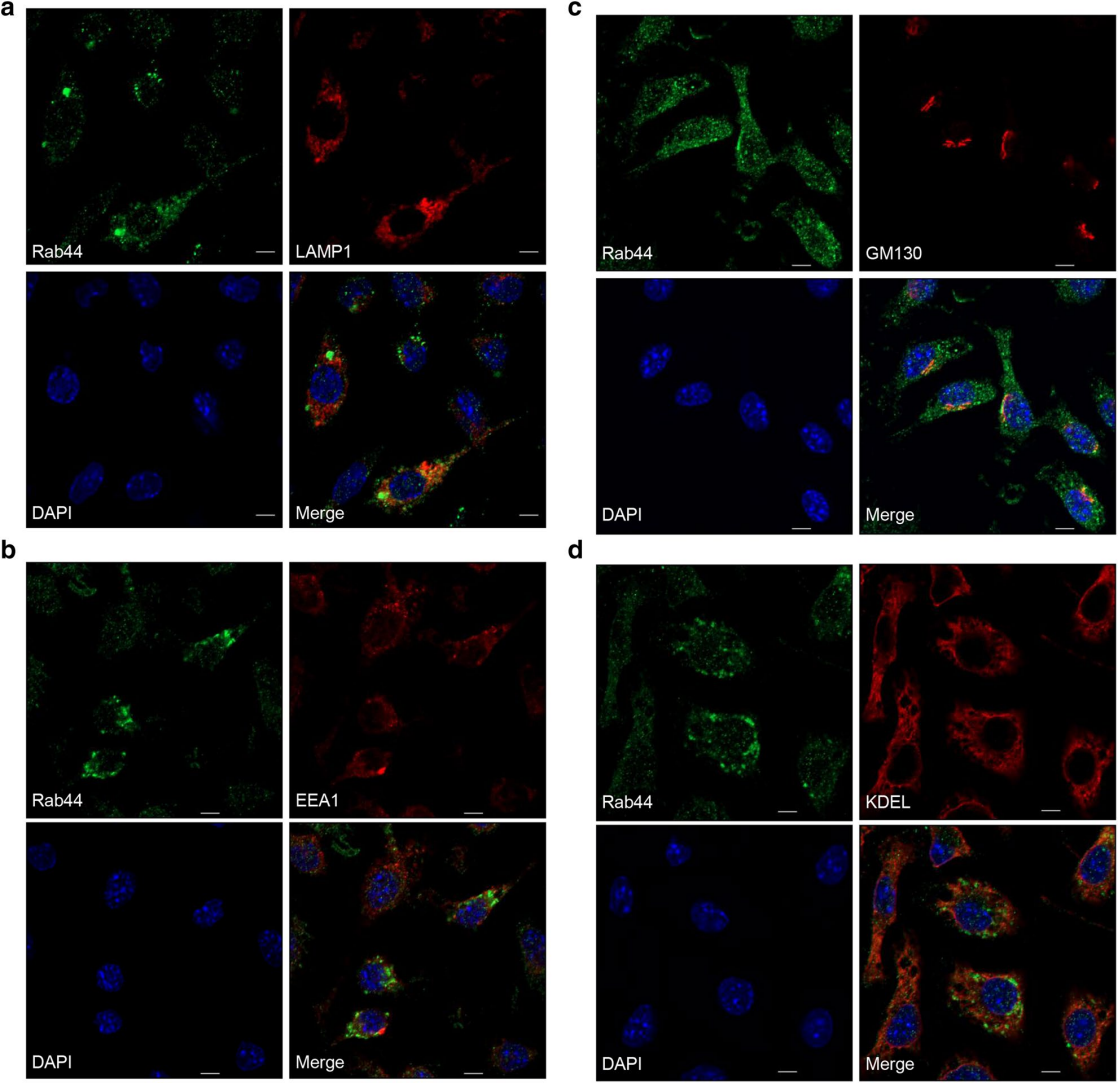
Immunofluorescence analysis of Rab44 and organelle markers in BMMs. The cells on glass coverslips were fixed, permeabilised with 0.1% Triton X-100 in PBS, and then allowed to react with antibodies against (**a**) LAMP1 (marker for late endosomes/lysosomes), (**b**) EEA1 (marker for early endosomes), (**c**) GM130 (marker for the Golgi complex), and (**d**) KDEL (marker for ER). After washing, the samples were incubated with a fluorescence-labelled secondary antibody and then visualised by confocal laser microscopy. Bar: 5 μm.

Our previous study showed that the short form of GFP-Rab44 fusion overexpressed in the murine monocytic cell line, RAW-D, is predominantly localised in the Golgi complex and lysosomes^9^. Therefore, we investigated the localisation of endogenous Rab44 in BMMs. As a result, Rab44 was localised to vesicle-like compartments, in which it was partially colocalised with LAMP1 (lysosome marker) (Fig. 5a), but not with EEA1 (early endosome marker), (Fig. 5b), GM130 (the Golgi marker) (Fig. 5c), and KDEL (endoplasmic reticulum marker) (Fig. 5d). Thus, endogenous Rab44 in BMMs was partially localised in lysosomes.

### The localisation of Rab44 in BMMs is changed by lipopolysaccharide (LPS) treatment

**Figure 6:**
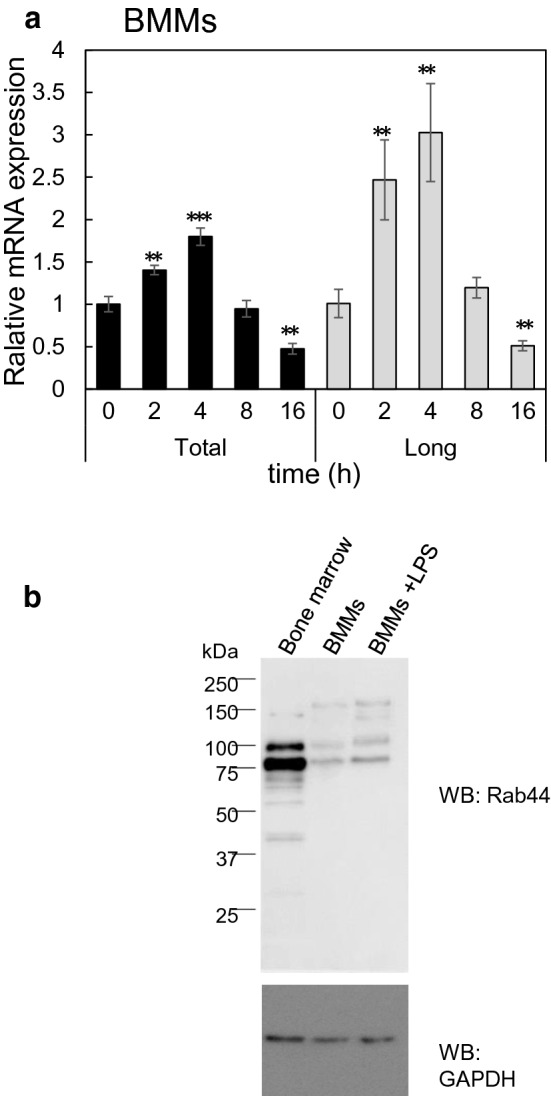
Effects of LPS on Rab44 expression in BMMs. (**a**) Quantitative RT-PCR determination of Rab44 mRNA expression in BMMs after stimulation with LPS (10 ng/mL) for the indicated time. The data are represented as the mean ± S.D. of values from three independent experiments. The asterisks indicate statistical significance compared to the control at time 0, **P < 0.01; ***P < 0.005. (**b**) Western blotting of Rab44 protein expression levels in bone marrow cells, BMMs, and LPS-stimulated BMMs. Cell lysates (of equal protein amounts) were subjected to SDS-PAGE followed by western blotting with antibodies against Rab44 and GAPDH (control).

Next, we examined whether Rab44 expression in BMMs is changed after stimulation with LPS. The total expression levels of long-form and short-form Rab44 reached a peak at 4 h after stimulation and then gradually decreased up to 16 h (Fig. 6a). Similarly, the expression pattern of long-form Rab44 also peaked at 4 h after stimulation (Fig. 6a). We confirmed the molecular weight of Rab44 expressed in BMMs compared with that in bone marrow cells; as shown in Fig. 6b, Rab44 proteins in the latter were mainly detected at multiple bands of 75 and 100 kDa. Multiple minor bands were also detected. In BMMs, the Rab44 proteins were detected at multiple bands of 75, 100, 140, and 150 kDa. Consistent with the mRNA level experiments, the Rab44 protein levels were all increased after LPS stimulation (Fig. 6b).

**Figure 7:**
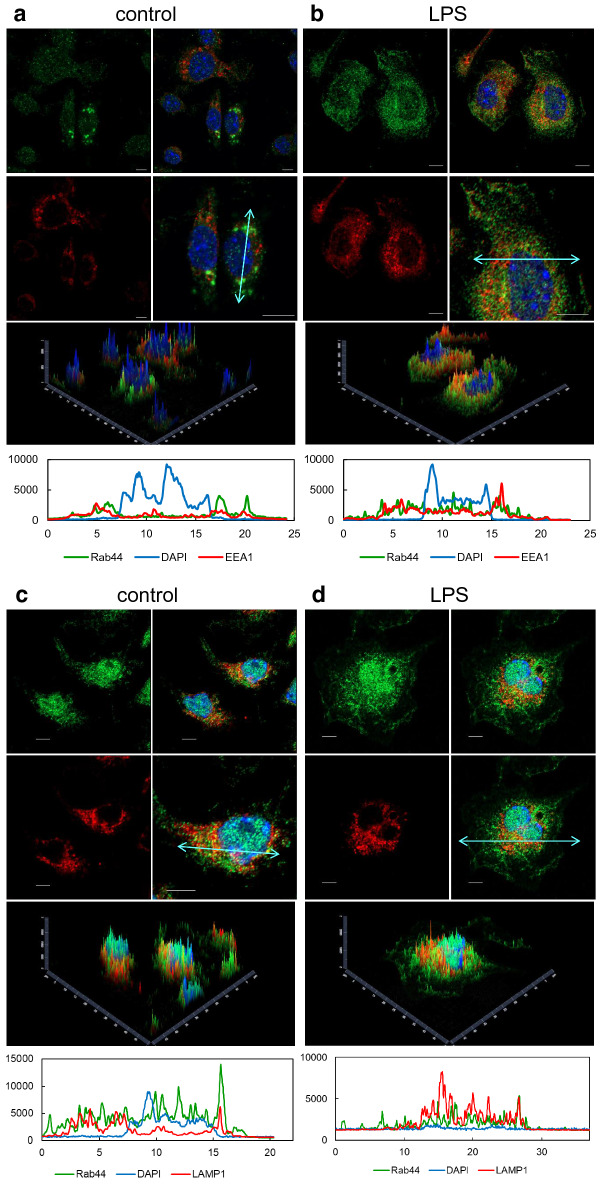
Effects of LPS on subcellular localisation of Rab44 in BMMs. The cells on glass coverslips were stimulated without (**a**, **c**) or with (**b**, **d**) LPS for 4 h, fixed, permeabilised with 0.1% Triton X-100 in PBS, and then allowed to react with antibodies against Rab44 and EEA1 (a marker for early endosomes) (**a**, **b**), and LAMP1 (**c**, **d**) (a marker for lysosomes). After washing, the samples were incubated with a fluorescence-labelled secondary antibody and then visualised by confocal laser microscopy. Bar: 5 μm.

We investigated whether the subcellular localisation of Rab44 in BMMs was altered upon LPS treatment. Without stimulation, Rab44 in BMMs was slightly colocalised with EEA1 (early endosome marker) (Fig. 7a, see Fig. 5b). Upon treatment with LPS, however, Rab44 was partially colocalised with EEA1 and partially detected in the plasma membrane (Fig. 7b). Moreover, Rab44 in BMMs was partially localised with LAMP1 (lysosome marker) (Fig. 7c, see Fig. 5a). Treatment with LPS hardly altered the colocalisation of Rab44 with LAMP1 (Fig. 7d). These results indicate that the localisation of Rab44 in BMMs is partially changed by LPS treatment.

### Rab44 expression levels are altered by treatment with immunomodulators

**Figure 8:**
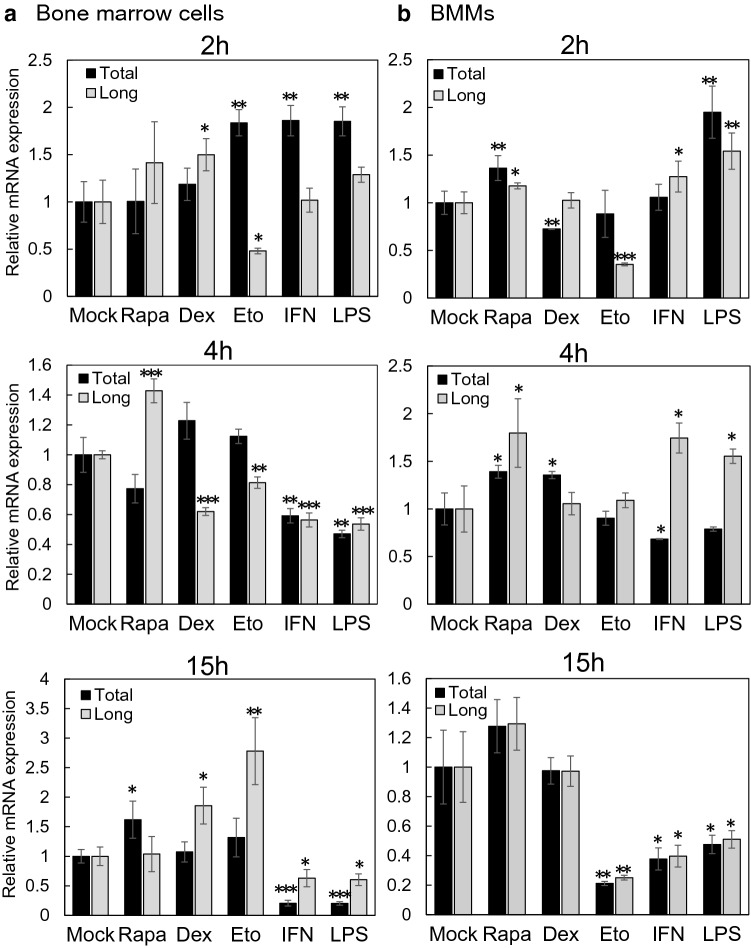
Effects of various immunomodulators on Rab44 expression levels in bone marrow cells and BMMs. Quantitative RT-PCR analysis of time course of Rab44 mRNA expression levels upon treatment with mock, rapamycin (Rapa), dexamethasone (Dex), etoposide (Eto), interferon (IFN)-γ, and lipopolysaccharide (LPS). (**a**) bone-marrow cells, (**b**) BMMs. The asterisks indicate statistical significance compared to the control (Mock), *P < 0.05; **P < 0.01; ***P < 0.005.

Finally, we examined whether Rab44 expression levels were affected by treatment with immunomodulators. We selected immunosuppressants, including rapamycin (Rapa), dexamethasone (Dex), and etoposide (Eto), and immunostimulants, such as interferon (IFN)-γ and LPS. The Rab44 expression was separately measured for the total expression of Rab44 and the expression of long-form Rab44 only. When bone-marrow cells were treated with various immunomodulators, the total levels of Rab44 and the long-form levels were distinctly altered (Fig. 8a). Upon treatment with Rapa and Dex, the total levels and long-form levels differently increased, although Dex treatment caused a transient decrease of the long-form amount after 4 h of treatment (Fig. 8a). Interestingly, Eto treatment led to an increase in the total level of Rab44, and a decrease in the long-form level at incubation time of 2 h, while it caused the opposite results after 15 h of treatment (Fig. 8a). Upon treatment with IFN and LPS, the time-course patterns were similar; the total levels of Rab44 were rapidly increased after 2 h of treatment, but they were significantly decreased at incubation time of 4 and 15 h (Fig. 8a). Under the same conditions, the level of long-form Rab44 was decreased after 4 and 15 h of treatment (Fig. 8a).

In contrast, when BMMs were treated with the same immunomodulators, the total levels of Rab44 and the levels of long-form Rab44 were similarly changed in spite of some exceptions (Fig. 8b). Rapa treatment caused an increase in both total levels and the long-form levels, Dex treatment had slight effects on both total levels and the long-form levels (Fig. 8b). Eto treatment caused decrease in the long-form levels after 2 h of treatment, and it reduced both total levels and the long-form levels (Fig. 8b). Upon treatment with IFN and LPS, the total amount after treatment with LPS was increased after the 2 h incubation period, and the long-form levels, after treatment with IFN and LPS at incubation time of 4 h were found to be increased, and subsequently all levels of both of them were decreased after 15 h of treatment (Fig. 8b). These results indicate that expression between total and long-form of Rab44 differently changed by various immunomodulators.

## Discussion

In this study, we examined the tissue distribution of Rab44 and found that it was highly expressed in bone marrow cells. Immunohistochemical analysis indicated that Rab44 was extensively expressed in CD117^+^ cells, but not Sca-1^+^ cells in the bone marrow. Moreover, the expression levels of Rab44 were changed during differentiation of immune-related cells and by treatment with immunomodulators. Importantly, the localisation of Rab44 in BMMs was influenced by LPS treatment. Thus, Rab44 expression and localisation in bone marrow cells and BMMs changes with cell differentiation and stimulation.

Rab44 was highly expressed in bone marrow cells and slightly expressed in the epididymis, lung, skin, spleen, thymus, ovary, uterus, and liver. In fact, Rab44 was detectable in only a small number of cells in the tissues. These results suggest that Rab44 is cell type-specific or tissue-specific and is probably expressed in immune-related cells, since in the bone marrow, Rab44 was detected in CD117^+^ cells, but not in Sca-1^+^ cells. Previously, CD117 was thought to be expressed in HSCs, multipotent progenitors (MPPs), oligopotent progenitors (OPPs), and lineage-restricted progenitors (LRPs)^11,12^. Given that Sca-1 was thought to be a marker for early-stage cells, such as HSCs and MPPs^13^, Rab44 may be expressed in OPPs, LRPs, and their related cells. This notion is based on the classical or revised hematopoietic hierarchy model, which describes a stepwise differentiation of HSCs via intermediate progenitors into mature cells. However, recent studies have suggested a continuous model that describes heterogeneity of HSCs gradually acquire well-balanced cell groups under physiological conditions^14,15^. In this model, there are no obvious boundaries between different hierarchical levels of hematopoietic cells^14^. Therefore, it is important to determine the exact mechanisms by which Rab44 is specifically expressed in CD117^+^ Sca-1^−^ cells in the bone marrow in further studies.

Rab44 expression was also characterised by lower expression levels in differentiated immune cells, such as NEUTs, BMMs, and DCs that are mature even in immune system cells. Moreover, the expression levels between total and long-form of Rab44 differently changed by various immunomodulators. In bone marrow cells, the total amounts of Rab44 and the amount of long-form Rab44 were distinctly altered. This difference is probably due to the expression levels of long form and short form between bone marrow cells and BMMs. Namely, the short forms were mainly expressed in bone marrow cells, whereas both the long and short forms were equivalently expressed in BMMs. However, the expression levels of various “small” Rab GTPases in monocyte-derived DCs after stimulation with LPS has been reported^16^. Early and recycling endosomal Rabs (Rab4b, 5a, 8a, 11a, 21, 35) exhibited an early increase after LPS activation, whereas late endosomal Rabs (Rab7a, 9a) did not. Similarly, when the majority of Rab members were examined for changes in expression levels in response to challenge by LPS in the mouse brain, Rab20 and Rab32 were upregulated during the acute phase of inflammation^17^. It is of interest to determine the transcriptional regulation of Rabs to modulate cell function during environmental differentiation.

The Rab44 proteins in the bone marrow were detected in two forms with molecular masses of approximately 75 and 100 kDa, while those from the spleen and thymus were approximately 150 kDa. The relationship between proteins of 75–100 kDa is probably due to splicing variants, such as the short and long forms. In fact, these numbers are consistent with the expected molecular weights. On the other hand, the difference between the two molecular masses could be due to the presence of monomers or dimers. Namely, the coiled-coil domain in Rab44 may be responsible for a possible dimer formation. Generally, coiled-coil proteins are known to form a homodimer^18^. For example, representative coiled proteins such as GM130 and EEA1, which all form homodimers, play an important role in membrane tethering. Western blot analysis and immunoprecipitation of EEA1 show that EEA1 is detected in two forms: a dimer and monomer^19^. Therefore, the higher molecular weight proteins may play different roles from those of lower molecular weights.

Endogenous Rab44 in BMMs was localised partially in lysosomes, but not in the Golgi complex. These results are slightly different from our previous results, which indicated that Rab44-GFP fusion overexpressed in the murine monocytic cell line RAW-D is predominantly localised in the Golgi complex and lysosomes^9^. The discrepancy may be due to the expression levels of Rab44, such as small amounts of endogenous Rab44 versus highly expressed exogenous Rab44. Alternatively, the endogenous Rab44 may have comprised a mixture of 100 kDa protein (long form) and 75 kDa protein (short form). Indeed, our group has found that the long and short forms of mouse Rab44 are differentially localised. When long-form mouse Rab44 is expressed in the rat basophilic leukaemia cell line RBL-2H3, it is mainly localised in the lysosomes. However, short-form mouse Rab44 is localised in the small vesicle-like compartments, which are distinct from lysosomes and early endosomes in RBL-2H3 cells. Thus, Rab44 appears to change intracellular localisation depending on the cell type, expression level, and long or short form.

In conclusion, Rab44 is highly expressed in bone marrow cells and is extensively detected in CD117^+^ cells, but not in Sca-1^+^ cells in the bone marrow. Moreover, the expression levels of Rab44 are changed during differentiation of immune-related cells and by treatment with immunomodulators.

## Methods

### Antibodies and reagents

Mouse monoclonal anti-GAPDH (Cat. No. M171-3), anti-EEA1 (Cat. No. M176-3), and anti-KDEL (Cat. No. M181-3) antibodies were from Medical and Biological Laboratories (Nagoya, Japan). Alexa Fluor 488-conjugated goat anti-rabbit IgG and Alexa Fluor 555-conjugated goat anti-mouse, anti-rat and anti-rabbit IgG were from ThermoFisher Scientific (Rockford, IL, USA). Rat monoclonal anti-LAMP1 (Cat. No. 553792) and mouse monoclonal anti-GM130 (Cat. No. 610823) were from BD Biosciences (Franklin Lakes, NJ, USA). Dexamethasone, etoposide, and IFN-γ were purchased from FUJIFILM Wako Pure Chemicals (Osaka, Japan); and rapamycin was from LC Laboratories (Boston, MA, USA). Other reagents, including the protease inhibitor cocktail and LPS, were purchased from Sigma Aldrich (Tokyo, Japan).

### Cell culture

Isolation of BMMs was carried out according to a previously described method^20^. Briefly, bone-marrow cells from mice femurs and tibias were cultured overnight in α-MEM containing 10% foetal bovine serum (FBS), penicillin (100 U/mL) and streptomycin (100 μg/mL) at 37 °C in 5% CO_2_. Non-adherent cells were harvested to a stroma-free bone marrow cell culture system containing 50 ng/mL M-CSF. After three days, the adherent cells were harvested as BMMs.

To develop DCs, BMMs (1 × 10^6^ cells/mL) were further incubated in RPMI 1,640 medium supplemented with 5% FBS, penicillin (100 U/mL), streptomycin (100 μg/mL), and 2-ME (50 μM) in the presence of GM-CSF (10 ng/mL) at 37 °C in a 5% CO_2_ atmosphere. At days 4 and 7 of culture, immature DCs loosely attached to the monolayer were further incubated by exchanging 1/3 of the volume with a new culture medium. For DC maturation, on  day 8, the cells were cultured in a cell culture dish and stimulated with LPS (100 ng/mL) for 18 h.

To isolate NEUTs, mice were injected peritoneally with 4% thioglycolate (2 mL/mouse). After 14 h, peritoneal exudate cells were isolated from the peritoneal cavity by washing with phosphate-buffered saline (PBS). The cells were incubated with RPMI 1,640 medium supplemented with 10% FBS, penicillin (50 U/mL), and streptomycin (50 g/mL) at 37 °C with 5% CO_2_. After incubation for 2 h, adherent cells were removed, and non-adherent cells were isolated as NEUTs.

THP-1 cells were purchased from American Type Culture Collection (Manassas, USA). The cells were cultured in complete RPMI 1640 medium containing 10% FBS, penicillin (50 U/mL), and streptomycin (50 g/mL). To induce monocyte/macrophage differentiation, THP-1 cells were added with 30 nM PMA and cultured 2 days in complete RPMI 1640 medium. PMA was then removed by aspiration and the adhesive cells were washed twice with RPMI 1640 medium.

To analyse the effects of immunomodulators on bone marrow cells and BMMs, cells were incubated in complete media supplemented with rapamycin (RAPA) (0.1 μM), dexamethasone (DEX) (0.1 μM), etoposide (ETO) (10 μM), interferon (IFN)-γ (20 ng/mL) or LPS (100 ng/mL) for 15 h.

### Immunofluorescence microscopy

The cells were cultured on glass inserts. The cells were fixed with 4.0% paraformaldehyde in PBS for 20 min at 25 °C, and then washed three times with PBS. After quenching with 50 mM NH_4_Cl for 10 min, the cells were permeabilised with 0.1% Triton X-100 in PBS for 4 min, washed with PBS three times. The cells were incubated with primary antibodies at 4 °C for 30 min after blocking with 0.2% gelatin in PBS. The cells were washed four times with 0.2% gelatin in PBS and then incubated with Alexa Fluor-conjugated secondary antibodies, followed by nuclear staining with DAPI. The samples were observed using a laser-scanning confocal microscopic imaging system (LSM800; Carl Zeiss, AG, Jena, Germany).

### Immunohistochemistry

Cryosections for immunohistochemistry were obtained from 8-week-old C57BL/6 mice. Tissue sections were fixed in 10% neutral buffered formalin for 10 min and then washed with PBS. Endogenous peroxidase was blocked with 0.3% H_2_O_2_ in methanol for 30 min, followed by incubation with G-Block (Genostaff, Tokyo, Japan) and avidin/biotin blocking kit (Vector Laboratories, Burlingame, CA, USA). The sections were incubated with anti-Rab44 rabbit polyclonal antibody at 4 °C overnight. After washing with TBS, sections were incubated with biotin-conjugated anti-rabbit Ig (Dako) for 30 min at room temperature, after which peroxidase-conjugated streptavidin (Nichirei, Tokyo, Japan) was added for 5 min. Peroxidase activity was visualised by diaminobenzidine. The sections were counterstained with Mayer's Hematoxylin (MUTO, Tokyo, Japan), dehydrated, and then mounted with Malinol (MUTO). For lung and spleen staining, however, Simplestain Mouse MAX-PO (R) (Nichirei) was used in place of the secondary antibody and endogenous peroxidase treatment. Immunofluorohistochemistry of mouse bone marrow was performed as described previously^20^. Briefly, 5 μm-thick femur sections were prepared from wild-type mice. Sections were blocked in PBS containing 5% normal donkey serum. Rabbit polyclonal anti-Rab44 IgG (1:1,000) was used as the first antibody, followed by fluorescent labelling with Alexa fluor 488-conjugated anti-rabbit IgG. Images were acquired using a LSM800 confocal scanning microscope (Carl Zeiss, AG, Jena, Germany) equipped with Airyscan, and the images were processed automatically with an additional manual adjustment of + 0.5 per channel using the Zeiss Zen Blue 2.3 software package.

### RT-PCR analysis

Total RNA was extracted from tissue or cells using TRIzol reagent (Invitrogen, Carlsbad, CA, USA). The cDNA was obtained by reverse transcription using Revertra Ace (Toyobo, Osaka, Japan). Quantitative RT-PCR was performed using Brilliant III Ultra-Fast SYBR Green QPCR Master Mix (Agilent Technology, La Jolla, CA, USA) by a LightCycler 480 (Roche Diagnostics, Mannheim, Germany). The following primer sets were used:

Mouse GAPDH, forward: AAATGGTGAAGGTCGGTGTG and reverse: TGAAGGGGTCGTTGATGG; mouse Rab44, total forward: AGAGACCACACACACTCTC and reverse: CTCCTGTAAGTCTGTTCTTG; long form forward: GGAAGAATTCAGCTCTGGAC and reverse: GGTGGAAGTCACAGATTCTC; human Rab44, forward: AGGCTTTCTGGCCAAGATGA and reverse: GGTCAGAGTCACGCTTCCTC; human GAPDH, forward: TCAAGGCTGAGAACGGGAAG and reverse: TGGACTCCACGACGTACTCA.

### Western blot analysis

Western blot analysis was performed as described previously^21^. Briefly, cells were rinsed twice with ice-cold PBS and lysed in PBS containing 0.1% Triton X, 1 mM PMSF, and proteinase inhibitor cocktail. The protein concentration was measured using BCA Protein Assay Reagent (Thermo Pierce, Rockford, IL, USA). An equal amount of protein was applied to each lane. After sodium dodecyl sulphate–polyacrylamide gel electrophoresis (SDS–PAGE), the proteins were electroblotted onto a polyvinylidene difluoride membrane. The blots were blocked with 5% nonfat milk solution containing Tris-buffered saline (TBS)/0.1% Tween 20 for 1 h at 25 °C, probed with various antibodies overnight at 4 °C, washed, incubated with horseradish peroxidase-conjugated secondary antibodies (Cell Signaling Technology, Danvers, MA, USA), and finally detected with ECL-Prime (GE Healthcare Life Sciences, Tokyo, Japan). The immunoreactive bands were analysed by LAS4000-mini (Fuji Photo Film, Tokyo, Japan).

### Statistical analysis

Quantitative data are presented as mean ± standard deviation (S.D.). Statistically analyses were performed using Prism 7 (GraphPad, San Diego, CA, USA). The unpaired *t*-test was used to identify differences between concentrations when a significant difference (*P < 0.05, **P < 0.01 or ***P < 0.005) was determined by analysis of variance.

## Supplementary information

Supplementary information.
