# Supplementary material for: Expression and localisation of Rab44 in immune-related cells change during cell differentiation and stimulation
Source: Sci Rep. 2020 Jul 1;10:10728. doi: 10.1038/s41598-020-67638-7 (PMC7329882; doi:10.1038/s41598-020-67638-7)
Supplement: Supplementary file 1 — Supplementary information. [file 41598_2020_67638_MOESM1_ESM.pdf]

Supplementary Information

Expression and localisation of Rab44 in immune-related cells change during cell differentiation and stimulation

Mitsuko Tokuhisa[1, 2, 3], Tomoko Kadowaki[1]*, Kohei Ogawa[1, 2, 3], Yu Yamaguchi[2], Mizuho Kido[4], Weiqi Gao[4], Masahiro Umeda[3], and Takayuki Tsukuba[2]*

[1]Department of Frontier Life Science, Graduate School of Biomedical Sciences, Nagasaki University, Sakamoto 1-7-1, Nagasaki 852-8588, Japan

[2]Department of Dental Pharmacology, Graduate School of Biomedical Sciences, Nagasaki University, Sakamoto 1-7-1, Nagasaki 852-8588, Japan

[3]Department of Clinical Oral Oncology, Nagasaki University Graduate School of Biomedical Sciences, Nagasaki, Sakamoto 1-7-1, Nagasaki 852-8588, Japan

[4]Department of Anatomy and Physiology, Faculty of Medicine, Saga University, Saga 849-8501, Japan

Contents

Supplementary Figures

Figure S1~S2: Original gel images of immunoblot analysis……………………………………3

Figure 1 c

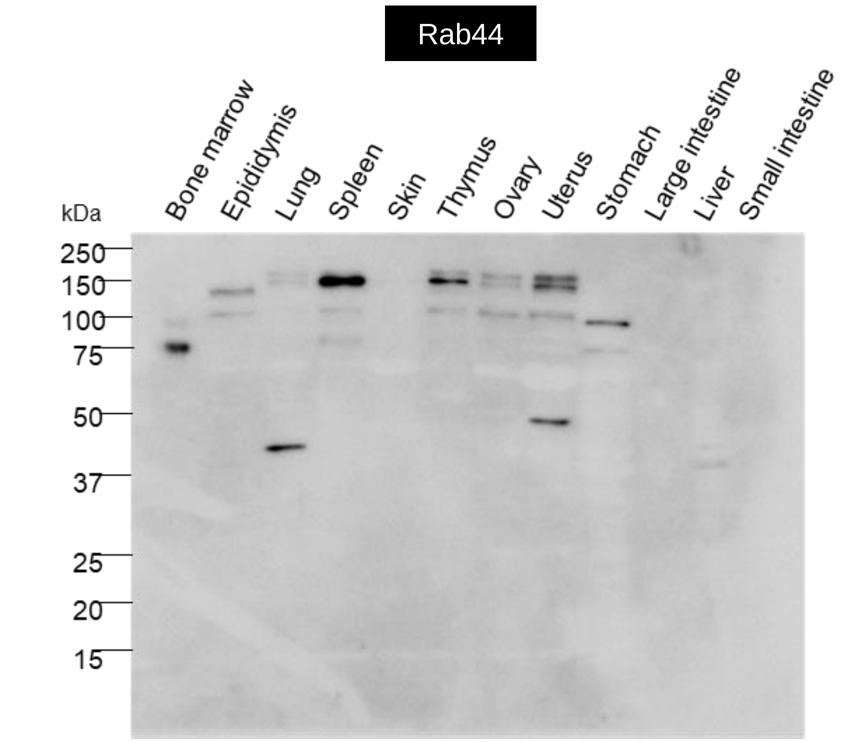
Rab44
Bone marrow
Epididymis
Lung
Spleen
Skin
Thymus
Ovary
Uterus
Stomach
Large intestine
Liver
Small intestine
kDa
250
150
100
75
50
37
25
20
15

Figure 6 b

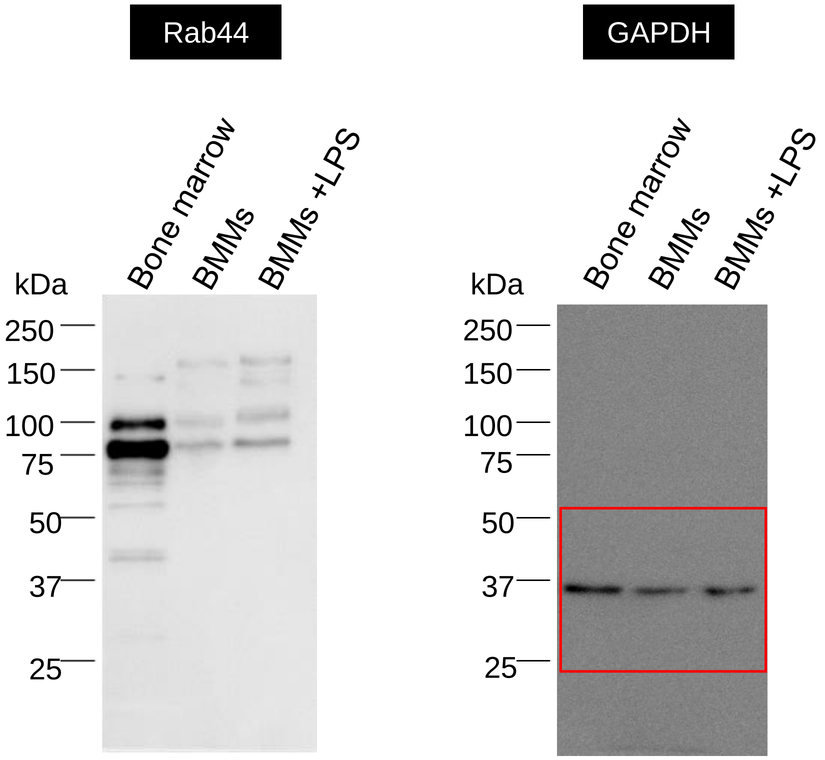
Rab44
GAPDH
Bone marrow
BMMs
BMMs +LPS
kDa
250
150
100
75
50
37
25
